# Adaptation of Better Conversations with Primary Progressive Aphasia to Norwegian

**DOI:** 10.3390/brainsci15090994

**Published:** 2025-09-15

**Authors:** Ingvild Winsnes, Monica Norvik, Anna Volkmer

**Affiliations:** 1 Department of Linguistics and Scandinavian Studies, University of Oslo, 0317 Oslo, Norway; monica.i.norvik@uit.no; 2 Department of Education, UiT The Arctic University of Norway, 9019 Tromsø, Norway; 3 Division of Psychology and Language Sciences, University College London, London WC1N 1PF, UK; a.volkmer.15@ucl.ac.uk

**Keywords:** primary progressive aphasia, communication partner training, cultural adaptation, conversation, dementia

## Abstract

Background/Objectives: People with primary progressive aphasia (PPA) and their communication partners report that having conversations becomes more difficult over time. They want speech and language therapy to help them have better conversations. Communication partner training has shown promise as an approach for people with PPA and their communication partners. However, there are currently no communication partner training programs available in Norwegian for people with PPA. The Better Conversations with Primary Progressive Aphasia (BCPPA) is a communication partner training program developed in the UK. In this study, we aimed to culturally adapt the BCPPA to meet the needs of Norwegian people with PPA. Methods: Guided by adaptation elements identified in a systematic review of frameworks for cultural adaptation, we translated the BCPPA into Norwegian before piloting it with four participant dyads, comprising people with PPA and their communication partners. The translated BCPPA was compared to the original BCPPA to identify adherence with core intervention components. Semi-structured interviews were used to explore the acceptability of the intervention to participant dyads. Outcome data, including Goal Attainment Scaling, coding of conversation behaviours from video samples, the Aphasia Impact Questionnaire, and the Communicative Effectiveness Index, were recorded pre-, post-, and three months after intervention delivery to explore outcomes for Norwegian participant dyads. We used the Framework for Reporting Adaptations and Modifications-Enhanced to document the modifications. Results: The results indicate high adherence to the core components in the original BCPPA. The pilot demonstrated that the participant dyads found the BCPPA acceptable, but they made some additional suggestions to complete the cultural adaptation further. Despite the progressive nature of PPA, the participant dyads achieved their goals on the Goal Attainment Scaling, and group analysis demonstrated maintenance on the Aphasia Impact Questionnaire and the Communicative Effectiveness Index over the three time points. Conclusions: This study demonstrates that the Norwegian version of the BCPPA was acceptable to the participants with PPA and their communication partner in this study. As the first communication partner training program for people with PPA and their communication partners in Norwegian, the BCPPA has the potential to be a valuable treatment tool to support people affected by PPA to have better conversations.

## 1. Introduction

Primary progressive aphasia (PPA) refers to a group of language-led dementias associated with frontotemporal lobe degeneration (FTLD) or Alzheimer’s disease pathology [1]. The prevalence is estimated to be 3–4 per 100,000 [1,2], which conservatively yields more than 200 diagnosed cases of PPA in Norway. At present, three variants of PPA have been described in the international consensus criteria for diagnosis. The semantic variant PPA (svPPA) and the non-fluent/agrammatic variant (nfvPPA) are most often associated with FTLD, whilst the logopenic variant (lvPPA) in most cases is associated with Alzheimer’s disease pathology [1]. The three variants of PPA each present with distinct language deficits. The semantic variant PPA (svPPA) is the best-described variant. It is characterised by anomia and difficulties in single-word comprehension. The non-fluent/agrammatic variant PPA (nfvPPA) is characterised by agrammatism, with omission of function words and inflections errors, effortful speech with disrupted prosody, speech sound errors, and reduced speech rate. The most recently described variant, logopenic variant PPA (lvPPA), is associated with word-finding difficulties and sentence repetition deficits, hypothesised to be caused by problems with verbal working memory [1,3].

PPA impacts both the person with PPA and the people who are close to them. Davies et al. [4] conducted a scoping review of studies exploring the communication needs of people with PPA (PwPPA) from the perspective of PwPPA, their family members, and speech and language therapists. They identified difficulties with conversation as an area of need for both the people with PPA and their family members. These findings are supported by the results of Volkmer et al.’s [5] international consensus study across 17 countries. In their study, people with PPA and their communication partner (CP) were asked what they most wanted to change about their lives with PPA. The results from the Norwegian participants demonstrated that they prioritised participating in conversations [6]. These results were almost universally agreed upon internationally, and people affected by PPA reported that they want to be able to participate in conversations with family and friends [5]. Collectively, this research emphasises the need for a focus on conversation in speech and language therapy with people affected by PPA.

Communication partner training (CPT) is a well-documented conversation-focused approach from the post-stroke literature [7,8]. (We use “training” in connection with the concept of communication partner training, as this is used in the literature [7,8]. “Therapy” is used when referring to speech and language therapy, or the therapy provided in the intervention.) Simmons-Mackie and Damico [9] found that building on existing strategies (e.g., using gestures) was a more effective method than learning new strategies for both post-stroke aphasia and progressive aphasia. Similarly, in their systematic review of functional communication interventions for PPA, Volkmer et al. [10] concluded that speech and language therapists (SLTs) should routinely involve a communication partner in therapy. Emphasis should be put on strengthening the current strategies the person with PPA is using.

At present, the only CPT program available in Norwegian is the Better Conversations with Aphasia (BCA), aimed at people with post-stroke aphasia. The BCA-Norsk is available at UCL Extend: Better Conversations with Aphasia [11] and is used by clinical SLTs in Norway. There is currently no CPT program in Norwegian tailored to the needs of people with PPA. Developed in the UK and based on the BCA, the Better Conversations with Primary Progressive Aphasia (BCPPA) is a manualised CPT program tailored to the needs of people with PPA and their CPs. The BCPPA was co-produced with PwPPA and comprises four sessions with home-based tasks between sessions [12]. A UK-based randomised controlled pilot study involving 18 dyads (a person with PPA and their CP) demonstrated that the BCPPA was acceptable to the participants as well as the SLTs delivering the intervention, with high treatment fidelity. In the intervention group, all self-reported outcome measures for the PwPPA and CPs changed in the intended direction, and 29 of the 30 goals set and rated by the participants using Goal Attainment Scaling were achieved or overachieved. When these goals were operationalised into observable behaviours, 18 of the 32 behaviours changed in the intended direction when pre- and post-video recordings were compared [13]. Given the urgent need for a PPA-specific CPT intervention for a Norwegian context, it seems logical that the BCPPA may be best suited for translation and cultural adaptation.

### Cultural Adaptation

With the increase in prevalence of dementia worldwide, there has been an increase in cultural adaptation of dementia interventions over recent years [14]. According to Bernal et al. [15], cultural adaptation is “the systematic modification of an evidence-based treatment (EBT) or intervention protocol to consider language, culture, and context in such a way that it is compatible with the client’s cultural patterns, meanings, and values” (p. 362). To promote transparency and replicability in cultural adaptations, a framework to guide the adaptation process and a framework to document the adaptation are recommended [14,15].

Several frameworks to guide the adaptation process have been developed. In a systematic review of frameworks for psychosocial dementia interventions, Day, Laver, Jeon, Radford, and Low [14] identified twelve cultural adaptation frameworks. They found that most frameworks involve modifications to both the content (e.g., translation, changes to examples or images used) and the context (e.g., format or setting). Eight of the frameworks reviewed by Day, Laver, Jeon, Radford, and Low [14] advocated for fidelity to the original intervention, either by retaining the core components of the intervention or by, after having identified them, purposefully adapting the key components to align with cultural aspects in the new population. Core components are defined as the primary components that are responsible for mediating therapeutic change [16]. Nine of the frameworks identified engagement, e.g., addressing facilitators and barriers to service, as a key element in adaptation. Notably, six frameworks emphasised cultural competence, including the cultural competence of the practitioners in activities such as adjusting the therapeutic approaches. These elements, which address factors to make the intervention more receptive and understandable to a new population, are considered peripheral components. It is suggested that they, in contrast to core components, do not affect the fidelity of the intervention, but rather enhance the acceptability of an intervention in a new population [16]. None of the cultural adaptation frameworks in Day, Laver, Jeon, Radford, and Low’s [14] review were specifically designed for dementia interventions. When undertaking cultural adaptations of dementia interventions, Day, Laver, Jeon, Radford, and Low [14] recommend considering the values and beliefs of the target population, the intervention, and the purpose of adaptation when choosing a framework to use.

Whilst there are no systematic reviews of cultural adaptation reporting frameworks, the Framework for Reporting Adaptations and Modifications-Enhanced (FRAME) is an example of a framework developed for documenting and reporting modifications to an intervention [17,18]. FRAME comprises eight aspects that aim at enhancing the documentation of modifications: (1) when and how the modifications occurred, (2) if the modification was planned or not, (3) who determined the modification, (4) what was modified, (5) at what level did the modification occur, (6) type or nature of modification, (7) fidelity to the original intervention, and (8) reason for modification [18]. FRAME has been used in a study to adapt a Danish CPT intervention for post-stroke aphasia to different cultures in the Global North and Global South [19], and to translate and culturally adapt dementia interventions from English-speaking American culture to the Hispanic/Latino population in America [20] and to the Korean population in America [21]. Since all these studies reported FRAME to be a useful framework for documenting modifications, we chose to use FRAME [19,20,21].

Informed by the adaptation elements identified by Day, Laver, Jeon, Radford, and Low [14] and employing FRAME to document these modifications, the ultimate goal of this study was to translate and pilot the BCPPA to inform the cultural adaptation of the BCPPA to meet the needs of people with PPA in Norway. The study aimed to answer the following research questions:

1. Can the BCPPA be adapted to a Norwegian population whilst retaining the core components of the original BCPPA intervention?
2. Is the BCPPA acceptable to Norwegian PwPPA and their CPs?
3. How can we measure whether the BCPPA has a positive impact on everyday conversations between Norwegian PwPPA and their CPs in terms of goal attainment, conversation behaviours, communication-related quality of life, and communication effectiveness?

## 2. Materials and Methods

The translation and piloting of the BCPPA-Norsk were led by the first author IW, a native Norwegian speaker, and conducted in collaboration with the last author AV, the developer of the original BCPPA, ensuring the necessary cultural knowledge to adapt an intervention culturally and fidelity to the original intervention [18,22]. As there is no adaptation framework specifically for dementia interventions, we drew upon the adaptation elements identified by Day, Laver, Jeon, Radford, and Low [14] for the adaptation process. To enhance the transparency of our adaptation, FRAME was used to document and report on modifications.

The study was approved by the Norwegian Agency for Shared Services in Education and Research (reference number 147255). It complies with the Declaration of Helsinki and the National Guidelines for Research Ethics in the Social Sciences and the Humanities. We obtained informed written consent from the PwPPA and CPs prior to enrolment in the study.

### 2.1. Better Conversations with Primary Progressive Aphasia

The BCPPA comprises four one-hour sessions, delivered by an SLT, and is aimed at the dyad (both the PwPPA and CP). Home-based tasks are encouraged between sessions and reviewed in the upcoming session. Each session focuses on a specific topic and has specific aims, as shown in Table 1.

Having made a video recording of their own conversations prior to therapy, the first session aims to provide the dyad with opportunities to explore what conversation is for them and what can go wrong in their conversations. In the second session, the dyad works on setting goals based on facilitators and barriers they have identified in their own conversation videos. Facilitators are communication behaviours that enhance the progressivity and intersubjectivity of the conversation, e.g., when a PwPPA uses gesture or description when they cannot produce the word they are thinking of verbally. Whilst barriers are communication behaviours that hinder progressivity and intersubjectivity, one example of this is when a CP uses a test question that they know the answer to, such as when a teacher or parent asks, e.g., “What is the colour of my top?” [when it is evident that the colour is known to both parties]. The third session focuses on practising strategies and problem-solving issues the dyad may have encountered when using strategies outside of therapy. The focus in the third session is continued in the last session; additionally, planning for future changes in communication is addressed [12,23].

### 2.2. Translation

**Figure 1:**
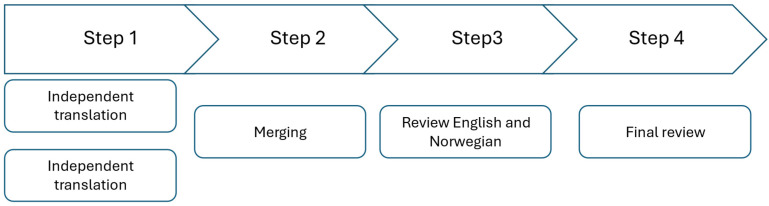
Overview of the translation process.

At the time of translating the BCPPA, the BCA had previously been adapted into Norwegian [11]. The BCPPA builds on the BCA. Some of the decisions around terminology were made based on the translation of the BCA to maintain consistency. For example, turn-taking (i.e., taking turns speaking) and strategies were chosen for this reason. Three experienced SLTs (Døli, Erikstad, and Winsnes), who were involved in the BCA adaptation, translated the BCPPA. Figure 1 shows an overview of the translation process. Erikstad and Winsnes translated the BCPPA independently (step 1) before meeting and discussing any disagreements. The disagreements were minor and related to how a word or sentence could be best translated, for example, whether to translate “set goals” directly or use “make goals”, both of which can be used in Norwegian. The two translated versions were merged into one; if they did not reach an agreement, their two alternatives were kept in the text (step 2). Døli, who is a simultaneous English/Norwegian bilingual, compared the original BCPPA with the translated merged version and made suggestions where Erikstad and Winsnes did not reach an agreement (step 3). Finally, Døli and Winsnes met and agreed on a version ready for piloting (step 4).

The Norwegian version was closely aligned with the original version. However, minor changes were made to certain components of the BCPPA to make it more culturally appropriate (see Appendix A for an overview). For example, some of the culture-specific examples were changed (e.g., cricket was replaced with handball), and information about where to seek help and support was changed to relevant organisations in Norway. Where the manual refers to a module that is part of the Better Conversations e-learning but not translated into Norwegian, we added a statement that this module is not available in Norwegian. This module is aimed at the SLTs. Given the high degree of English proficiency in Norway [24], we decided that referring the SLTs to the English version was sufficient. The changes made during the translation were considered to be peripheral components, thus not affecting the fidelity of the treatment. They were all planned and decided upon by the research team and related to changes in content. Two of the changes were made to improve fit with recipients and address cultural factors.

### 2.3. Fidelity to Core Components

We measured fidelity to core components using the treatment fidelity checklist. This checklist was developed to measure treatment fidelity in the UK-based BCPPA pilot RCT [23]. The core components fidelity checklist can be found in Appendix B. As we wanted to measure the fidelity of the translation and adaptations of core components in the Norwegian BCPPA, IW compared the Norwegian session plans and handouts to the core components fidelity checklist. For each component from the checklist that was present in the session plans and handouts, 1 point was given, and 0 points were given if not present. For example, the component “SLT provides an overview of the BCPPA therapy” from the fidelity checklist was considered present in the translated session plans and handouts when it was stated that the SLT should provide an overview of the therapy and what it comprised.

### 2.4. Pilot

The pilot aimed to explore the acceptability of the translated BCPPA program for further adjustments before testing the intervention in a clinical setting. To inform a future international multi-centre effectiveness study, we also aimed to explore the best way to measure changes for Norwegian dyads that receive the BCPPA.

#### 2.4.1. Participants

Participants were recruited from the Memory Clinic at the Oslo University Hospital and a geriatric outpatient ward at a local hospital. Potential participants were provided with written information about the project from their treating medical clinician. If potential participants consented to be contacted by IW, their telephone number and information on whether they preferred to be contacted by text message or phone were passed to her. IW contacted participants and invited them to discuss the study further, to assess them against the inclusion and exclusion criteria, and to obtain their consent for the study.

PwPPA were included if they had been diagnosed with PPA as per Gorno-Tempini, Hillis, Weintraub, Kertesz, Mendez, Cappa, Ogar, Rohrer, Black, Boeve, Manes, Dronkers, Vandenberghe, Rascovsky, Patterson, Miller, Knopman, Hodges, Mesulam, and Grossman’s [3] international consensus criteria, had a CP (e.g., family member or friend) who was able to participate in the study with them, were able to give informed consent, and were able to receive the treatment in Norwegian. They were excluded if they did not meet one or more of the inclusion criteria or had cognitive difficulties or other conditions (e.g., severe somatic or psychiatric disorder) that made attendance difficult. The CPs in the study were recruited through the PwPPA, who were asked to identify a person close to them with whom they talked regularly.

To establish an overview of language and cognitive function in the participants with PPA, *the Comprehensive Aphasia Test—Norwegian version* (CAT-N) [25] and the Mini-Mental State Examination—Norwegian *revised* (MMSE-NR3) [26] were administered pre-intervention. The CAT-N, MMSE-NR3, and AIQ-21 were administered by IW in the socio-cognitive lab at the University of Oslo. The CPs answered the CETI without a researcher present, but were given the opportunity to ask questions if needed. GAS was completed as part of the BCPPA.

The BCPPA intervention was delivered in person by the first author, IW, an experienced SLT, who received training in delivering the BCPPA. The intervention was primarily provided in the socio-cognitive lab at the University of Oslo. However, if needed, it could also be provided in the participant’s home.

#### 2.4.2. Acceptability

To explore the acceptability of the BCPPA to the participants, we explored recruitment and retention data. We also conducted semi-structured interviews individually with each participant immediately after the intervention. Semi-structured interviews are considered an appropriate method for exploring acceptability [27]. The interviews comprised questions related to the format, the translation, and cultural appropriateness (see Appendix C). The interviews lasted between 6 and 22 min and were audio recorded.

#### 2.4.3. Measuring Change for Norwegian Dyads Receiving the BCPPA

Table 2 provides an overview of the measures, the rationale for using them, and at which time point they were administered.

The dyads completed Goal Attainment Scaling (GAS) as part of the intervention. GAS is a method to measure individual goals in a standardised way; it yields a T-score with a mean of 50 [28], and a T-score between 40–60 has in previous research been considered to be in the expected outcome range [29,30]. In the BCPPA, the dyads identify their own goals and evaluate them in the last session. The goals can be related to the PwPPA, the CP, or both. These goals are then targeted in the intervention, and at the end of the intervention, the dyads are asked to rate whether they had achieved or not achieved the goals on a five-point scale [13].

Video recordings of conversation samples were made in the participants’ homes with no researcher present. The dyads could choose to borrow a video camera from the University of Oslo or download the Visio app to their smartphones. The Visio app is a secure video recording application that sends data directly to Services for Sensitive Data, a secure storage facility at the University of Oslo. The dyads were instructed to make at least four recordings, each lasting at least 10 min, and to record in situations where they normally would have a conversation (e.g., dinner time). They were given a list of topics to talk about, which can be downloaded from Better Conversations Downloads, UCL Faculty of Brain Sciences [31], if they found it difficult to come up with something themselves.

For social and functional communication, we used the Norwegian version of the Aphasia Impact Questionnaire-21 (AIQ-21) [25] and the Norwegian version of the Communicative Effectiveness Index (CETI) [32]. The AIQ-21 is a patient-reported outcome measure developed for measuring the impact of aphasia on communication, participation, and well-being [25]. It was the favoured measure in the UK-based BCPPA pilot study [13] and is available in Norwegian. The CETI measures changes in functional communication as perceived by a communication partner [32]. The CETI was not used in the UK-based study; however, it is the measure available in Norwegian that is closest to the Communication Confidence Rating Scale for Aphasia, which was used in the Volkmer, Walton, Swinburn, Spector, Warren, and Beeke [13] study.

#### 2.4.4. Analysis

For acceptability, recruitment and retention data were analysed using descriptive statistics. The interview data were transcribed verbatim by IW using NVivo 14 and analysed using qualitative content analysis, following the steps outlined in Erlingsson and Brysiewicz [33]. We chose this approach as it enabled us to systematically analyse and summarise our results, and answer questions about why, how, in what way, or by what means [33]. In line with this approach, IW familiarised herself with the data by reading and re-reading the transcripts. She then grouped the text into meaning units, which were condensed and coded before being categorised and grouped into themes. The analysis was conducted in Norwegian. Table 3 provides a translated example of the analysis process from meaning unit to theme.

Measures: Data were analysed in R Studio using R version 4.2.3 (15 March 2023 ucrt)—“Shortstop Beagle.” Raw scores from the CAT-N, MMSE, AIQ, and CETI were analysed using descriptive statistics. Given the small sample size, we used the nonparametric Friedman’s test to explore any change on a group level. To analyse the GAS, we followed the procedure outlined in Turner-Stokes [28]. The goals were weighted, and, as recommended, baseline scores were rated as −1.

To assess whether there was a change in the frequency of the targeted behaviour identified by the participants using GAS, the goals were linked to an observable behaviour from the enactment measure developed by Volkmer, Beeke, Warren, Spector, and Walton [23]. This measure comprises 27 observable behaviours, where 16 relate to the PwPPA and 11 to the CP. Twenty-three of the behaviours are defined as facilitators and four as barriers, meaning behaviours that either enhance or halt the progressivity and intersubjectivity of the conversation [23]. We refer the reader to Volkmer, Beeke, Warren, Spector, and Walton [23] for a full description of the enactment measure. Two dyads identified goals related to clarifying what the other person had said. As there is no corresponding code to this in the enactment measure from the Volkmer, Beeke, Warren, Spector, and Walton [23] study, we added an additional facilitator behaviour “Understanding check-CP paraphrases the meaning of PwPPA’s prior turn as a means of checking what they have said”, which was taken from Best et al.’s [34] study in conversation therapy for people with aphasia and their CPs. To prepare the video recordings for analysis, a five-minute video clip was obtained from each recording following the procedure outlined in Best, Maxim, Heilemann, Beckley, Johnson, Edwards, Howard, and Beeke [34] and applied in the UK-based pilot study of the BCPPA [13,23]. In line with Best, Maxim, Heilemann, Beckley, Johnson, Edwards, Howard, and Beeke [34] and Volkmer, Walton, Swinburn, Spector, Warren, and Beeke [13], the five-minute clip was taken from the second half of the 10-min recordings to ensure the participants had acclimatised to the recording device and the interaction was as naturalistic as possible. For each dyad, the mean value from the video recordings at each time point was used in the analysis. If data were missing from a time point, the average from an equal number of recordings at each time point was used (see Table 4). In cases where we had to exclude any recordings, we excluded the last one.

IW and a research assistant received training from AV on how to code conversation behaviours in the videos using a sample video clip from the BCPPA study [13]. Four video clips (10%) were randomly selected from the sample and transcribed, and all potential behaviours (from the identified 28 behaviours) were coded by both IW and the research assistant. IW then compared the coding before a consensus meeting was held with AV, and any discrepancies were discussed and agreed on. The video clips for the current study were then randomised, and the research assistant coded all 28 behaviours. This ensured the research assistant was masked to the time points and behaviours targeted in therapy. Finally, the research assistant coded the remaining data for all 28 behaviours. The goals from the GAS were then compared to a corresponding code.

## 3. Results

### 3.1. Acceptability of the Norwegian BCPPA

As we found a 100% fidelity to core components in the Norwegian version of the BCPPA, we can assume that this version is equivalent to the original.

#### 3.1.1. Recruitment and Retention

Eight PwPPA consented to be contacted by IW. Two PwPPA were excluded because they did not meet the inclusion criteria; one did not have a CP who was able to participate in the study, and one was unable to receive the treatment in Norwegian. Two people declined to participate, stating that they did not believe speech and language therapy of any kind to be useful for them. Four dyads met the inclusion criteria and consented to participate. All participants were retained for the duration of the study and completed all intervention sessions. Dyads 2 and 3 had to pause the intervention for, respectively, 7 weeks between the third and fourth sessions and 4 weeks between the first and second sessions, due to illness, but both returned to complete the intervention.

All four included participants had received a diagnosis of PPA within the last year prior to enrolment in the study. Table 5 provides an overview of participants’ demographic characteristics and language profiles based on pre-intervention assessment of linguistic skills. As shown in Table 5, there were two bilingual dyads in the study. Dyad 2 previously spoke German and Norwegian at home, but after the diagnosis, they mainly spoke Norwegian, using only some German words and expressions. Dyad 4 spoke English and Norwegian at home and had previously lived in English-speaking countries; they also had children under the age of 18, with whom they primarily communicated in English.

#### 3.1.2. Content Analysis of Semi-Structured Interviews

Following the procedure outlined in Erlingsson and Brysiewicz [33] we identified five themes from the interview data: (1) awareness of how communication works, (2) communication strategies, (3) the amount of work, (4) unfamiliar terminology, and (5) the BCPPA provides a different perspective.

1. Awareness of how communication works: The CPs emphasised increased awareness of how they communicate and how communication works in general:
  “To become more aware of it, to analyse what is happening in the conversation, that well, to become more aware” (CP3);
2. Communication strategies: The PwPPA and CPs highlighted identifying and practising communication strategies as one of the most useful aspects of the BCPPA. One of the participants described how she had started describing a word when encountering a word search:
  “And then I have this lovely talking around talking (…) before I could not say it, but now I can talk around” (PPA1);
3. The amount of work: Even though all of the participants reported the BCPPA to be useful and that they would do it again, some reported it was hard to find time to do home-based tasks:
  “It helps [home-based tasks] because you have to just do it, deal with things right now.” (PPA1) “yes, yes, yes home-based tasks can be a bit difficult (…) it’s always something happening but it is useful so we try to do it even though we don’t always manage to.” (CP1).
  For some participants, sessions every week were too intensive, and they suggested a more distributed schedule but with more sessions, “and that it was not that often, but maybe one more time and then with more time between” (CP3). Others found four weekly sessions acceptable, saying “it was okay” (PPA4);
4. Unfamiliar terminology: The participants expressed uncertainty about some of the terminology:
  “You use this professional language that we don’t know. I think you should use normal language” (CP2).
  “I mean intonation (…) and turn-taking I think you can do that [the translation] a bit differently.” (PPA1);
5. The BCPPA provides a different perspective: The CPs reported that the BCPPA intervention made them realise that they had to change their own communication behaviour.
  “When things are difficult and we [as a couple] work well enough together, even if we don’t talk all the time, right? It gets very quiet after a while and then you get used to it (…) so I think it has been very good to participate” (CP3).
  They also pointed out that the BCPPA offered a different perspective on PPA “because it’s everything medical, the geriatrician was amazing, he had a lot of time for us, but the how it affects us in everyday life. That it is difficulties with communication, how yes what we can do, techniques and such” (CP4), and further, how it provided a place for talking about how PPA impacts life. “Kind of holding the space for us to confront those kinds of painful realities” (CP4).

### 3.2. Measuring the Impact of BCPPA on Everyday Conversations Between Norwegian PwPPA and Their CPs

#### 3.2.1. Goal Attainment Scaling

**Figure 2:**
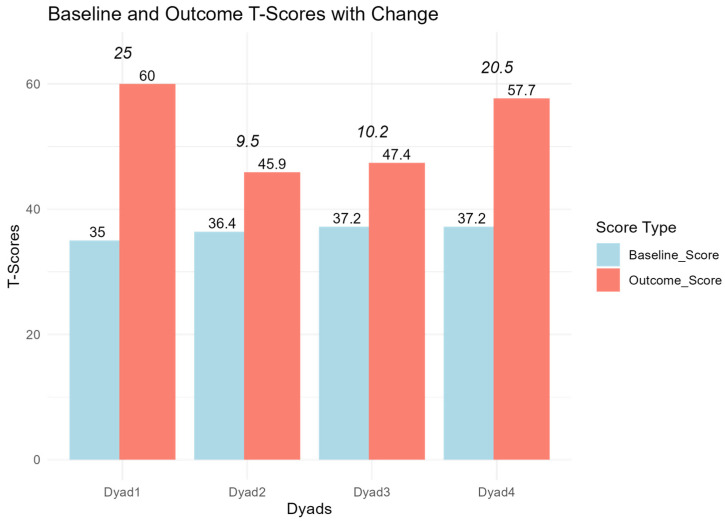
Results from Goal Attainment Scaling, with baseline score, outcome score, and change score (in italics).

Each dyad had between three and five goals. Some of them were specific to either the person with PPA or CP, and others were goals shared by the dyad. All goals from each dyad were used in the analysis. As shown in Table 5, three goals were achieved less than expected, four were achieved as expected, and seven were achieved more than expected. The attainment scores for all dyads fell within the expected outcome range (i.e., scores from 40–60) [29] and showed a change in the intended direction for all participants, as shown in Figure 2.

#### 3.2.2. Conversation Behaviours

**Figure 3:**
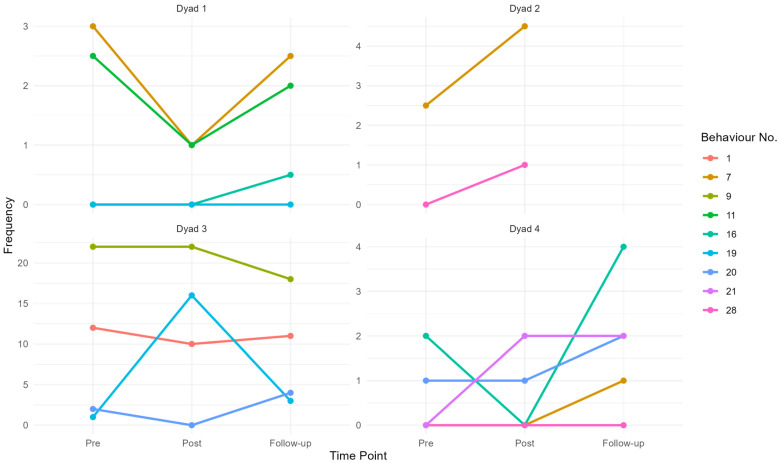
Frequencies of conversation behaviour per dyad at each time point.

The goals from the GAS were operationalised into observable communication behaviours, and linked to a conversation behaviour code from the enactment measure developed by Volkmer, Beeke, Warren, Spector, and Walton [23]. Eleven behaviours were linked with a behaviour code; in total, fifteen behaviour codes were applied. Not all goals could be operationalised into observable behaviour (e.g., “maintain what we got”). Additionally, not all behaviours corresponded to an existing behaviour code, and some behaviours were linked to more than one code. An overview of each goal, along with the dyads’ ratings of them, the corresponding behaviour, and the frequency observed in video analysis pre-intervention, post-intervention, and at follow-up can be found in Appendix D. Figure 3 illustrates the frequency of conversation behaviours per dyad at each time point (i.e., goals from GAS linked to an observable conversation behaviour). As all the conversation behaviours were facilitators, an increase in the frequency of the behaviour indicates improvement.

All behaviours were facilitators. At post-testing, four of the behaviours had changed in the intended direction, seven were the same, and three had changed in the unintended direction. At the three-month follow-up, we found a change in the intended direction when comparing follow-up to both pre- and post-testing, and when comparing post-testing to follow-up. That is, for five of the behaviours, the frequency of the behaviour was higher at both post-testing and follow-up. Three of the behaviours, which had changed in the unintended direction from pre-testing to post-testing, had at follow-up changed in the intended direction, with a higher frequency compared to post-testing, but not pre-testing. Two of the behaviours were the same at each time point, and one was maintained from post-testing to follow-up.

#### 3.2.3. Functional Self-Rating Measure

**Figure 4:**
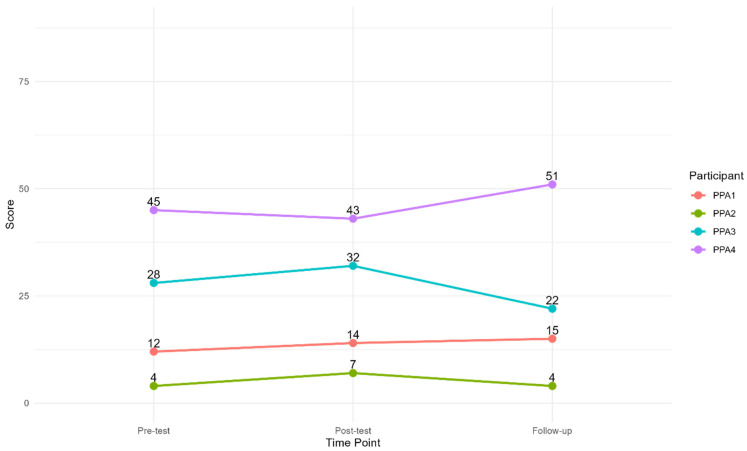
Individual scores on the AIQ from each time point. Note: A low score indicates the aphasia has less impact.

Immediately post-test, the AIQ scores increased for PPA1, 2, and 3, indicating an increase in the impact of PPA on the participants’ lives. At the three-month follow-up, the score had increased further for PPA1 but decreased for PPA2, indicating a lesser impact of PPA, and also decreased to below pre-testing for PPA3. In comparison, PPA4’s score decreased at post-testing, indicating the PPA had less impact on his life, but increased at follow-up, indicating that the PPA impacted his life more. Figure 4 displays the results.

**Figure 5:**
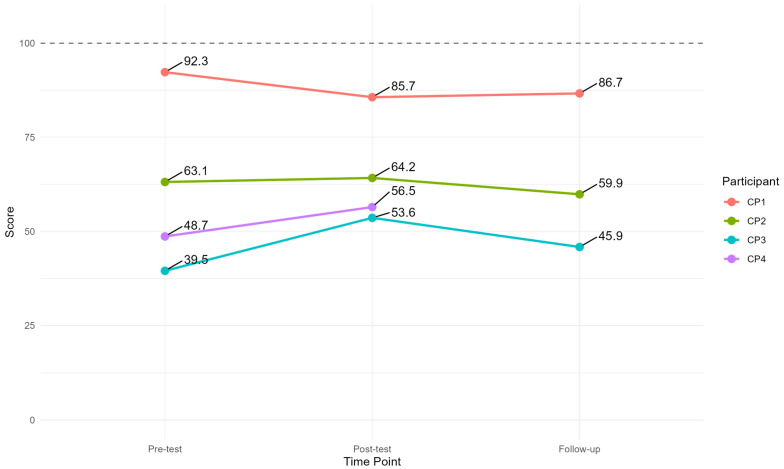
Results from the Communicative Effectiveness Index.

The CETI (Figure 5) showed that CP1 rated PPA1’s functional communication abilities almost at the ceiling at pre-testing, and there was a slight decrease at post-testing and follow-up. The scores from CP2 were relatively stable at all three test points, whilst CP3 rated PPA3’s functional communication abilities 14.07 points higher post-treatment, which, according to Lomas et al. [36], is a clinically significant change in the intended direction. At follow-up, the score had decreased but was higher than at pre-testing. Finally, the scores from CP4 improved from pre-testing to post-testing. The 3-month follow-up CETI data for CP4 are missing, as despite several requests to the CP, they were not returned.

When analysed as a group (Table 6), the descriptive statistics show that the scores on the AIQ-21 were maintained at approximately the same level at each time point. A Friedman’s test showed no statistically significant difference between the three time points, χ^2^ (2) = 0.93, *p* = 0.63.

At the group level (Table 7), the descriptive statistics show that the CETI scores were maintained at approximately the same level at each time point, with a slight improvement from pre-testing to post-testing. Friedman’s test showed no statistically significant difference between the three time points, χ^2^ (2) = 0.67, *p* = 0.72. Note that Friedman’s test requires data from each time point; thus, CP4 is omitted from the test.

## 4. Discussion

This exploratory study describes the cultural adaptation of the BCPPA through a process of translation and piloting in a Norwegian context. This is the first cultural adaptation of a communication partner training intervention for people with PPA in Norway. The Norwegian adaptation of the BCPPA is an important step in addressing what people with PPA and their care partners in Norway have identified as a treatment priority [5,6].

### 4.1. Fidelity to the Original BCPPA

The BCPPA-Norsk had a high fidelity to the original BCPPA, with 100% adherence to the core components of the intervention. Given the cultural similarities between the UK and Norway—both are Western industrialised countries with highly educated populations—it is perhaps not surprising that adherence to core components was high. For example, making and viewing video recordings, which are one core component, was considered to be appropriate also in Norway, whereas in other cultures, this may not be appropriate. There were, however, some peripheral changes made during the translation process to improve the fit with the recipients and to enhance the receptiveness of the BCPPA to the Norwegian population [16,37], such as changing examples of conversation topics when practising strategies.

### 4.2. Acceptability to Norwegian PwPPA and CPs

The BCPPA-Norsk was acceptable to the PwPPA and CPs in our study. There were no dropouts, and despite having to pause the intervention because of illness, Dyad 2 and Dyad 3 returned to complete the intervention, indicating a high level of engagement with the intervention. In the interview, the participants reported the intervention to be useful, emphasising that the BCPPA offered a new perspective and that the focus on conversation and their own communication was especially helpful. However, the need for some additional changes in peripheral components was highlighted, such as changes in terminology related to words that can be considered more technical (e.g., “intonation”). The use of unfamiliar terminology may have impacted how the intervention was received. For example, using “how your voice rises and falls when speaking” might make more sense to the participants than “intonation”. Language has been reported to be a factor to consider in adaptation work. It may impact how the intervention is received, and it is recommended to use terminology that is acceptable to the culture into which the intervention is adapted [16,37]. The participants also reported different needs related to dosage and intensity of the intervention; for example, CP3 reported that she would have liked a higher dosage (i.e., five sessions), with a more distributed schedule (i.e., every second week), whilst PPA4 reported that the dosage and intensity were acceptable. The context of delivery was reported as an important factor for CP4, emphasising that it was only because they could receive the treatment at home that they were able to participate in the study. Day, Laver, Jeon, Radford, and Low [14] identified in their systematic review that cultural adaptations should address engagement, such as awareness and access to the intervention. Culture competence was also identified as an adaptation element, which includes individualised therapeutic accommodations. It may be that a schedule tailored to the individual’s needs, while still adhering to the core components, is more appropriate than a standard model of delivery. The opportunity to choose where to receive the intervention also seems like an important factor when considering individualised therapeutic accommodations. Given that there has been no research exploring the issue, future work to understand context, dosage, and intensity for the delivery of communication partner training interventions, such as the BCPPA, for people with PPA will be important.

Whilst the preliminary results from this study show that the BCPPA was acceptable to the participants, only four of the eight potential dyads were recruited. The current study did not explore the reasons that the remaining dyads did not wish to participate, nor did we explore any referral barriers with the SLTs and neurologists at the collaborating centres. However, one reason may be a lack of awareness of speech and language therapy and how this can help. Previous research has shown that people affected by PPA and their families are not familiar with the breadth of interventions in speech and language therapy and may believe that nothing can be done to help [38]. There is an urgent need to better understand the barriers to accessing support so we are better able to raise awareness of the intervention in relevant communities [14].

This study demonstrated that measuring the impact of the BCPPA on everyday conversations between Norwegian PwPPA and their CPs is multifaceted. The dyads rated their goals as achieved, and the attainment scores from GAS indicated that the dyads’ goals were realistic; that is, the goals were not too easy nor too difficult. Collaborative goal setting and evaluation promote a client-centred approach and have been reported to enhance the participants’ motivation, their understanding of their own contribution to achieving the goals, and to set more realistic goals [39]. The collaborative goal setting that is central to the BCPPA intervention may have contributed to the realistic and achievable goals. The results suggest that GAS was a suitable measure in our study. This is in line with the results from Fegter et al.’s [40] systematic review and meta-analysis of GAS as an outcome measure across different dementia populations and interventions, which suggested that GAS is a suitable measure for this population and that it is able to measure functional change in areas such as communication. All other measures demonstrated maintenance. Coding of video-recorded conversations demonstrated that target behaviours were maintained across each time point. However, not all goals from the GAS could be operationalised into observable behaviours on the coding of video-recorded conversations, and not all behaviours were present as behavioural codes in the measure; thus, some goals were excluded from our analysis. Friedman’s test did not show any statistically significant difference on the AIQ-21. The descriptive analysis indicates that the participants’ scores on the AIQ-21 remained stable across each time point, both at the individual and group levels. Interestingly, the two participants with the highest scores (i.e., who experienced the most impact of the PPA) are the two who had nfvPPA and the highest score on the MMSE-NR3. It has been reported in the literature that people with nfvPPA initially are less affected by behavioural and cognitive impairments than people with lvPPA and svPPA [41]. It may be that the participants with nfvPPA in the current study had more awareness of their PPA and how it impacted them in their daily lives. As with the AIQ-21, we did not find any statistically significant differences on the CETI, and descriptive analysis showed that the results were relatively stable across time points except for Dyad 3, where the results showed there was a clinically significant change in functional communication for PPA3 as rated by his CP at post-testing. This was also the dyad with the lowest pretest score. Developed for post-stroke aphasia, it may be that the AIQ-21 and CETI, given the progressive nature of PPA, do not capture the needs of PwPPA and their CPs.

Given the small sample size, the results must be interpreted with caution and as preliminary. Based on the data from this study, we found that the ratings on the outcome measures overall were maintained, and all goals on the GAS were achieved. By showing maintenance at three-month follow-up, this study extends on the UK-based study, which showed maintenance immediately post-intervention [13]. It may be, given the progressive nature of the disease, that measuring the impact of the BCPPA-Norsk should focus on maintenance rather than improvement, which may in many cases be the aim of treatment for PwPPA [42]. Another way of measuring the clinical relevance of the outcome is to measure the minimal clinically important difference (MCID), which is defined as the smallest change between two scores that is subjectively meaningful to patients [43]. It was not possible to use this approach to inform the exploration of outcome data, as an MCID has not previously been established for any of the measures used in this study.

### 4.3. Limitations and Future Research

Whilst this was the first study to explore the use of the BCPPA-Norsk, the intervention was delivered in a research setting, by the same researcher who translated the intervention and analysed the study results. This may bias our results. Future research should address this by having assessment, intervention, and analysis undertaken by analysts and interventionists who are independent from one another. This exploratory study was limited by its small and heterogeneous sample in terms of PPA variant and the CP’s language background. Given these limitations, this small heterogeneous pilot is not representative of eligibility in real life, and a future larger trial, embedded within the clinical setting, will be important to provide more detailed information to inform implementation. It should also be noted that we do not have data on the onset of symptoms, which means that we do not know when in the course of the disease the intervention is best received. This study has highlighted that the terminology choices may impact acceptability and that adaptation is an iterative process. Future research on the BCPPA-Norsk will include adjustments to terminology based on participant feedback (e.g., “how your voice rises and falls” instead of “intonation”).

Future research should focus on the context, dosage, and intensity of CPT for PwPPA and their CPs, including possible differences between the needs of PwPPA and those of the CPs. Due to the low prevalence of PPA, reaching a large enough sample to conduct a randomised controlled trial (RCT) in Norway seems unlikely. Thus, there is a need for collaboration between countries, and an international multi-centre RCT may be one way to achieve a large enough sample and provide data to inform the effectiveness and applicability of the BCPPA across diverse contexts. This would also allow for the exploration of CPT candidacy among people with different types of PPA and various stages of the disease. A first step would be to develop outcome measures for PwPPA and their CPs, both patient-reported outcome measures and measures of communication behaviour. How to best measure change in communication, from a cross-cultural and cross-linguistic perspective, may be addressed by exploring common communication behaviours across languages and cultures.

## 5. Conclusions

This study demonstrates that the translated BCPPA-Norsk was acceptable to PwPPA and their CPs in this study. The participants found the intervention to be useful, but they also provided some additional suggestions to complete the cultural adaptation. Following the current pilot, we will address the suggestions made by the participants. This would be a first step to enhancing the Norwegian BCPPA. Further testing of the effectiveness of the BCPPA in an international, large-scale, multi-centre study would add to the evidence base of cross-cultural treatment for PwPPA and CPs. As the first CPT program for PwPPA and their CPs in Norwegian, the BCPPA has the potential to be a valuable treatment option.

## Figures and Tables

**Table 1:** Session overviews, aims, and adaptations at a glance. Table adapted from Volkmer et al. [23].

Session	Aims	Adaptations at a Glance
What is conversation?	Discuss the aims of therapy. Discuss and explore what conversation is and how it can go wrong. Initial viewing of their own conversation video.	Added an explanation in the session plan about an e-learning module that is not available in Norwegian.
Goal setting	Identify barriers and facilitators in their own conversation. Set goals for therapy based on this discussion.	Added an explanation in the session plan about an e-learning module that is not available in Norwegian.
Practice	Practice conversation using the strategies identified during goal-setting. Problem-solve any issues that have arisen in using identified strategies in conversations outside of therapy sessions.	Added an explanation in the session plan about an e-learning module that is not available in Norwegian. Changed some of the examples of conversation topics in the session plan.
Problem-solving and planning for the future	Practice conversation using the strategies identified during goal-setting. Consider planning for future changes in communication.	Changed information in the session plan about where to seek help and find more information about the diagnosis.

**Table 2:** Overview of outcome measures with rationale and time point.

Respondent	Measure	Rationale	Time Point
PwPPA	CAT-N	Overview of language function	Pre-intervention
PwPPA	MMSE-NR3	Overview of cognitive function	Pre-intervention
PwPPA	Aphasia Impact Questionnaire-21	Measure the impact of the PPA as perceived by the person with PPA.	Pre- and post-intervention, and at 3-month follow-up
CP	Communicative Effectiveness Index	Measure changes in functional communication as perceived by a CP.	Pre- and post-intervention, and at 3-month follow-up
Dyad	Goal Attainment Scaling	To individualise targets for intervention and measure change as rated by the dyads.	During intervention
	10-min video recordings	Measure change in communication behaviour targeted in treatment.	Pre- and post-intervention, and at 3-month follow-up

Note: 3-month follow-up refers to three months after post-intervention testing.

**Table 3:** Example of analysis process.

Meaning Unit	Condensed Meaning Unit	Code	Category	Theme
“Because you are there and kind of holding the space for us to confront those kinds of painful realities and to think about how we well kind of address it more directly and how we can handle the communication and stuff like that”	A space to confront the realities	Address realities	BCPPA as something different	BCPPA provides a different perspective

**Table 4:** Video clips from each time point from the dyads and the number used in the analysis.

Dyad	Pre-Testing	Post-Testing	Follow-Up	Video Clips from Each Time Point Used in the Analysis
1	2	2	2	2
2	2	2	0	2
3	2	2	1	1
4	1	1	1	1

**Table 5:** Participants’ demographic characteristics and language profiles.

	PPA1	PPA2	PPA3	PPA4
Age (years)	80	80	77	55
Biological sex	Female	Female	Male	Male
Diagnosis at the time of inclusion	Mixed	Mixed	nfvPPA	nfvPPA
Education (years)	18	13	14	18
Time post diagnosis (years)	<1	<1	<1	<1
First language	Norwegian	Norwegian	Norwegian	Norwegian
MMSE-NR3				
*Orientation (10)*	6	9	8	9
*Immediate recall (3)*	3	3	3	3
*Calculation (5)*	5	1	5	5
*Delayed recall (3)*	1	2	3	2
*Language and praxis (8)*	5	8	8	8
*Figure copying (1)*	1	1	1	1
Total MMSE Score (total possible score = 30)	21	24	28	28
CAT-N				
*Auditory comprehension (66)*	62	64	59	59
*Reading comprehension (62)*	57	53	52	56
*Repetition (74)*	66	66	59	53
*Naming (79)*	28	71	63	57
*Oral reading (70)*	70	69	70	70
Communication partner				
	CP1	CP2	CP3	CP4
Relationship to person with PPA	Husband	Husband	Wife	Wife
First language	Norwegian	German	Norwegian	English

Note: MMSE-NR3 = Mini Mental State Examination-Norwegian Revised 3, CAT-N = Comprehensive Aphasia Test–Norwegian, total possible score in parentheses. The education level of the PwPPA is representative for the Norwegian population, where approximately 76% have more than 13 years of education [35].

**Table 6:** Descriptive statistics for the Aphasia Impact Questionnaire-21 on the group level.

Timepoint	Mean	Median	SD
Pre	22.25	20	18.15
Post	24	23	16.47
Follow-up	23	18.5	20.08

**Table 7:** Descriptive statistics on the group level from the CETI.

Timepoint	Mean	Median	SD
Pre	60.91	55.89	23.08
Post	64.98	60.33	14.5
Follow-up	64.3	59.86	20.73

## Data Availability

The data presented in this study are available on request from the corresponding author, The data are not publicly available due to privacy restrictions.

## Abbreviations

The following abbreviations are used in this manuscript:

PPA	Primary progressive aphasia
PwPPA	People with primary progressive aphasia
CP	Communication partner
BCPPA	Better Conversations with Primary Progressive Aphasia
FTLD	Frontotemporal lobe degeneration
svPPA	Semantic variant PPA
nfvPPA	Non-fluent/agrammatic variant PPA
lvPPA	Logopenic variant PPA
CPT	Communication partner training
BCA	Better Conversations with Aphasia
UCL	University College London
SLT	Speech and language therapist
GAS	Goal Attainment Scaling
EBT	Evidence-based treatment
FRAME	Framework for Reporting Adaptations and Modifications-Enhanced
CAT-N	Comprehensive Aphasia Test-Norwegian version
MMSE-NR3	Mini-Mental State Examination-Norwegian Revised
AIQ-21	Aphasia Impact Questionnaire-21
CETI	Communicative Effectiveness Index

## Appendix A

Overview of adaptation/modifications elements.

**Table A1:** Overview of adaptation/modification of BCPPA.

	Process						Rationale		
Adaptation/Modification	When?	Planned?	Who Decided?	What Modified?	Content Modification	Fidelity Consistent?	Goal of Modification	Recipient	Additional Comments
Translation from English to Norwegian	Pre-implementation/planning/pilot	Planned/Proactive adaption	Research team	Content	Tailoring/tweaking/refining	Fidelity consistent/core elements or functions preserved	Improve fit with recipient	First/spoken languages	
Session plan 1: the word “therapy” was translated to “samtaletrening” (conversation training)	Pre-implementation/planning/pilot	Planned/Proactive adaption	Research team	Content	Tailoring/tweaking/refining	Fidelity consistent/core elements or functions preserved	Improve fit with recipient	First/spoken languages	“Therapy” is not used in relation to speech and language therapy in Norwegian. Speech and language therapy = “logopedi”, speech and language therapist = “logoped”.
Session plan 1: “Therapy map” was translated to “BCPPA-mappen” (the BCPPA map).	Pre-implementation/planning/pilot	Planned/Proactive adaption	Research team	Content	Tailoring/tweaking/refining	Fidelity consistent/core elements or functions preserved	Improve fit with recipient	First/spoken languages	
Session plan 1: Added “this module is not available in Norwegian” when the session plan refers to video samples.	Pre-implementation/planning/pilot	Planned/Proactive adaption	Research team	Content	N/A	Fidelity consistent/core elements or functions preserved	N/A		
Session plan 2: Added “This module is not available in Norwegian” when the session plan refers to Module 4 what to target in therapy	Pre-implementation/planning/pilot	Planned/Proactive adaption	Research team	Content	N/A	Fidelity consistent/core elements or functions preserved	N/A		
Session plan 3: Added “This module is not available in Norwegian” when the session plan refers to Module 4 what to target in therapy	Pre-implementation/planning/pilot	Planned/Proactive adaption	Research team	Content	N/A	Fidelity consistent/core elements or functions preserved	N/A		
Session plan 3: changed examples of sports (3e) and games (3k).	Pre-implementation/planning/pilot	Planned/Proactive adaption	Research team	Content	Tailoring/tweaking/refining	Fidelity consistent/core elements or functions preserved	Improve fit with recipients	First/spoken languages	Used common sports and games in Norway (e.g., handball instead of cricket).
Session plan 4: Information about where to seek help and more information about the diagnosis was changed to organisations and services in Norway.	Pre-implementation/planning/pilot	Planned/Proactive adaption	Research team	Content	Tailoring/tweaking/refining	Fidelity consistent/core elements or functions preserved	Improve fit with recipients	Access to resources	

## Appendix B. Core Component Checklist

**Table A2:** Session 1.

BCPPA CORE COMPONENT FIDELITY CHECKLIST: SESSION 1 What is Conversation?		
Component Number	Component	Present
1	SLT provides an overview of BCPPA therapy	1
2	SLT introduces aims of current session	1
3	SLT facilitates a discussion with dyad about their current understanding of conversation to support their learning	1
4	SLT provides Module 5.0 Handout 1: *How Does Conversation Work?*	1
5	SLT explains how conversation works using Handout 1: *How Does Conversation Work?*	1
6	SLT facilitates a discussion with dyad about how conversation may be affected by PPA	1
7	SLT provides Module 5.0 Handout 2: *What Can Go Wrong in Conversations?*	1
8	SLT explains Module 5.0 Handout 2: *What Can Go Wrong in Conversations?*	1
Sum		100

**Table A3:** Session 2.

BCPPA CORE COMPONENT FIDELITY CHECKLIST: SESSION 2 Goal Setting		
Component Number	Component	Present
1	SLT introduces aims of current session	1
2	SLT explains rationale for showing particular video clip	1
3	SLT shows dyad 2–3 pre-selected videos (30 s–2 min)	1
4	Each video clip is repeated 2–3 times (may not be applicable if they understood it the first time)	1
5	SLT ensures dyad has Handout 1: *How does Conversation Work?* provided in Session 1: What is Conversation?	1
6	SLT encourages CP and PwPPA to identify their own conversation facilitators in clip	1
7	SLT encourages CP and PwPPA to identify their own conversation barriers in clip	1
8	SLT provides Module 5.0 Handout 3: *Goal Setting*	1
9	SLT explains Module 5.0 Handout 3: *Goal Setting*	1
10	SLT supports PwPPA to identify their own therapy goals	1
11	SLT supports CP to identify their own therapy goals	1
12	Therapy goals were set for PwPPA	1
13	Therapy goals were set for CP	1
Sum		100

**Table A4:** Session 3.

BCPPA CORE COMPONENT FIDELITY CHECKLIST: SESSION 3 Practice		
Component Number	Component	Present
1	SLT introduces aims of current session	1
2	SLT asks dyad to identify 1–2 conversation strategies to work on in the therapy session	1
3	SLT facilitates role-play task to practise target conversation strategies	1
4	SLT supports dyad to agree conversation topic for role play	1
5	SLT facilitates discussion with dyad to review conversation strategy/strategies practiced in role play tasks	
6	SLT supports dyad to identify contexts (times and place) to practise conversation strategy/strategies for home-based task	1
7	SLT supports dyad to discuss any concerns regarding practicing conversation strategy/strategies at home	1
Sum		100

**Table A5:** Session 4.

BCPPA CORE COMPONENT FIDELITY CHECKLIST: SESSION 4 *Problem-Solving and Planning for the Future*		
Component Number	Component	Present
1	SLT introduces aims of current session	1
2	SLT supports dyad to agree conversation topic for role play	1
3	SLT facilitates role-play task to practise target conversation strategies	1
4	SLT facilitates discussion with dyad to review conversation strategy/strategies practiced in role play tasks	1
5	SLT ensures dyads has the Module 5.0 Handout 3: *Goal Setting*	1
6	SLT facilitates recap of the therapy goals set with CP and PwPPA in session 2	1
7	SLT asks dyad to identify whether they have achieved their therapy goals	1
8	SLT facilitates discussion with dyad about considerations for managing changes in future communication	1
9	SLT provides Module 5.0 Handout 5: *Information for the Future*	1
10	SLT explains Module 5.0 Handout 5: *Information for the Future* and sign-posts dyad to resources for the future	1
Sum		100

## Appendix C

BCPPA interview guide

Note that the questions were in Norwegian and provided to the participants with picture support.

1. What do you think was most useful?
2. What do you think should be changed?
3. What do you think about the organisation of the intervention?
4. Number of sessions?
5. Duration of each session?
6. Home-based tasks?
7. Are there any changes to the wording or language we should make?
8. The BCPPA was developed in the UK. Are there any changes we should make for it to work better in Norway?
9. Are there any changes that should be made to make the therapy even more adapted to the Norwegian context?

## Appendix D

Results from Goal Attainment Scaling and linked conversation behaviour.

**Table A6:** Results from Goal Attainment Scaling and linked conversation behaviour.

Dyad	Goals (Allocated to Either PwPPA or CP)	Achievement	Corresponding Behaviour No. and Description	Pre-Intervention Frequency	Post-Intervention Frequency	Follow-Up Frequency
1	Give PwPPA time when she speaks (CP)	+1	7: CP lets the conversation continue, i.e., pauses for further clues/so PwPPA can use strategies.	3	1	2.5
	Give myself enough time when speaking (PwPPA)	+1	16: CP asks “what do you want to talk about” or similar.	0	0	0.5
	Use gestures to describe a word when I cannot think of it (PwPPA)	+1	11: PwPPA talks around a word or concept when encounters word finding difficulties in a turn.	2.5	1	2
	Use objects when it is difficult to say the word (PwPPA)	0	19: PwPPA uses gesture or mime in a turn when encountering a word finding difficulty.	0	0	0
	Do one thing at a time; avoid distractions during conversations with my partner (CP)	0				
2	Maintain what we got (PwPPA/CP) ^b^	−1	N/A			
	Give enough time in conversation to let the other person talk (PwPPA/CP)	0	7: CP lets the conversation continue, i.e., pauses for further clues/so PwPPA can use strategies.	2.5	4.5	N/A
	Checking if we have understood each other correctly—clarifying what the other person means (PwPPA/CP)	0	28: Understanding check-CP paraphrases the meaning of PwPPA’s prior turn as a means of checking what they have said.	0	1	N/A
3	Express thoughts ^a^ (PwPPA)	−1	1: PwPPA responds with a substantive turn when asked a question.	12	10	11
			9: PwPPA initiates (verbal) turn.	22	22	18
	PwPPA signals when he needs help finding a word ^a^ (PwPPA)	+1	19: PwPPA uses gesture or mime in a turn when encountering a word finding difficulty	1	16	3
			20: PwPPA uses gesture or mime in a turn.	2	0	4
	Talk on the phone: PwPPA answers when CP calls, and CP remembers to pause to give PwPPA time to talk (PwPPA/CP) ^b^	+1	N/A			
4	Be patient, understanding, and allowing time in conversations (CP)	+1	7: CP lets the conversation continue, i.e., pauses for further clues/so PwPPA can use strategies.	0	0	1
			16: CP asks “what do you want to talk about” or similar.	2	0	4
	Repeating what PwPPA said to check if CP has understood him correctly (CP)	+1	28: Understanding check-CP paraphrases the meaning of PwPPA’s prior turn as a means of checking what they have said.	0	0	0
	CP use gestures to make it easier for PwPPA to understand, and PwPPA use gestures to be understood ^a^ (PwPPA/CP)	−1	20: PwPPA uses gesture or mime in a turn.	1	1	2
			21 CP uses gesture in turn.	0	2	2

Note: 0 = achieves goal as expected, −1 = less than expected, −2 = much less than expected, +1 = more than expected, +2 = much more than expected. ^a^ = The goal is linked to two behaviour codes. ^b^ = The goal could not be operationalised into a behaviour code.
